# Assessing the accuracy and efficiency of an electronic platform for managing childhood illnesses in rural China: A cluster randomized controlled trial

**DOI:** 10.1371/journal.pone.0352583

**Published:** 2026-09-15

**Authors:** Puhong Zhang, Yang Zhao, Jie Shang, Duolikun Nadila, Tao Li, Yu Liu, Gulizat Abdurixit, Jane Elizabeth Hirst

**Affiliations:** 1 The George Institute for Global Health, China Office, Beijing, China; 2 Duke-NUS Medical School, National University of Singapore, Singapore, Singapore; 3 Child Health Big Data Research Center, Capital Institute of Paediatrics, Beijing, China; 4 School of Computer Science and Engineering, Beihang University, Beijing, China; 5 Xinjiang Programme, Save the Children, Xinjiang, China; 6 Nuffield Department of Women’s & Reproductive Health, University of Oxford, Oxford, United Kingdom; 7 The George Institute for Global Health, Imperial College London, London, United Kingdom; The Chinese University of Hong Kong, HONG KONG

## Abstract

**Objectives:**

The Integrated Management of Childhood Illness (IMCI) faces challenges in capacity building and quality control. This trial aims to assess an electronic IMCI (eIMCI) platform in improving the effectiveness and efficiency in disease classification and management by community health workers (CHWs).

**Design:**

Cluster randomized controlled trial.

**Setting:**

Rural western China.

**Participants:**

24 CHWs and 72 ill children aged 2 months to 5 years (3 children per CHW). CHWs were randomly assigned to intervention or control groups.

**Interventions:**

The intervention CHWs received online training and performed disease management using the eIMCI platform featuring integrated training modules and decision-support tools. The control group received traditional face-to-face training and used paper-based IMCI protocols.

**Main outcome measures:**

Proportion of children correctly diagnosed or classified by CHWs, as determined by a pediatric specialist. Relative risk (RR) between groups was estimated using Poisson Generalized Linear Mixed Models incorporating a random intercept for CHW to account for clustering of children within individual CHWs and adjusting for key covariates at both the CHW and child levels.

**Results:**

The intervention group (13 CHWs, 39 children) had a higher rate of correct classification (64.1%) compared to the control group (11 CHWs, 33 children) (39.4%, P = .056). Multivariable regression analysis confirmed this (RR = 2.1, 95% CI: 1.5–3.1; P < .001). No significant difference was found in correct treatment rates (38.5% vs. 27.3%, P = .316). Online training reduced time and costs by approximately 80%, though with a slight decrease in post-training evaluation scores.

**Conclusions:**

The eIMCI platform shows potential in enhancing IMCI implementation and significantly reducing the training burden in resource-limited settings.

**Trial registration:** Chinese Clinical Trial Registry: ChiCTR2100042533, https://www.chictr.org.cn/showproj.html?proj=119995.

## Introduction

Despite a 50% drop in child mortality since 1990, the global under-five mortality rate was 38 per 1,000 live births in 2019, highlighting the need to accelerate progress toward the SDG target of 25 or fewer [1]. In 2018, under-five mortality rates in eastern, central, and western China were 4.2, 7.2, and 12.7 per 1,000 live births, respectively [2]. Western China faces particular challenges in delivering quality healthcare to children in its vast, sparsely populated rural areas.

The WHO and UNICEF introduced the Integrated Management of Childhood Illness (IMCI) strategy in 1995 to improve child survival through evidence-based interventions [3]. Over 100 countries have adopted IMCI, focusing on health worker guidelines for assessing and treating sick children, preventive care, and caregiver counselling [4,5]. Key elements include immunizations, antimicrobial use, and malnutrition advice [6]. While a 2012–2013 review confirmed IMCI’s effectiveness [7], its performance has been suboptimal due to low adherence, driven by high training costs, health worker illiteracy, lack of political support, and fragmented health systems [8–16]. Paper-based algorithms are especially challenging in busy clinics or when children present multiple symptoms, complicating diagnosis [17,18]. We faced similar challenges with the conventional approach. Although it improved accurate disease classification from 20% to over 60%, the resource-intensive training and supervision required were unsustainable.

Innovative solutions are urgently needed to improve diagnostic assessments and child healthcare management. Electronic IMCI (eIMCI) has shown promise in Africa and South Asia, enhancing adherence to protocols, reducing unnecessary antibiotics, and ensuring timely referrals [17,19–25]. However, few eIMCI tools include training modules or have been evaluated for accuracy and treatment effectiveness [24–26]. None have been developed or tested in China.

Based on a small trial, this study evaluates an eIMCI system with training modules against the paper-based approach, focusing on disease classification accuracy, training efficiency, and management practices in rural western China.

## Methods

### Study design

We conducted a small open-label, parallel cluster randomized controlled trial with a 1:1 allocation ratio. A ‘cluster’ was defined as a community health worker (CHW) assigned three child patients. All participating CHWs and child caretakers provided written informed consent. This study adheres to the CONSORT 2010 guidelines for cluster randomized trials.

### Study setting

The study took place in a rural county in western China, where CHWs, trained in primary care and basic medicine, provide essential health services to children aged 0–6 years [27,28]. Critically ill patients are referred to higher-level hospitals. Fieldwork occurred from December 2020 to February 2021. Due to COVID-19, evaluations shifted from primary care settings to a local city hospital, with patients selected from the same population typically managed by CHWs.

### Selection and randomization of CHWs

All qualified CHWs in the study county were invited to participate. They were randomly assigned to either: (i) traditional 4-day face-to-face training for paper-based IMCI, or (ii) online training for eIMCI. Post-training, 12 CHWs from each group were randomly selected, each assigned three eligible paediatric patients. Randomization was conducted centrally using simple randomization methods.

### Selection of children

Eligible children were aged 2 months to 5 years, exhibited symptoms of common diseases (e.g., pneumonia, diarrhea, fever, upper respiratory infections, diarrhoea, mastoiditis, ear disease, dysentery, measles, low body weight, and anaemia), and had parental consent. Exclusions included cases where IMCI visits might worsen the condition. Children were recruited from the hospital’s pediatric ward and outpatient clinic. Hospital staff and parents were instructed not to disclose prior diagnoses or treatments to CHWs.

### Interventions

Both groups were trained in IMCI procedures: assessment, disease classification, treatment, referral, caregiver advice, and follow-up [7].

The intervention group used the eIMCI system, comprising an online training module and a clinical decision-support platform. The system, developed for WeChat, included electronic documents, videos, and interactive features. CHWs completed the training at their own pace within one month, with progress contingent on answering embedded questions correctly. The decision-support platform guided CHWs through patient assessments, automatically generating disease classifications, treatments, and follow-up recommendations. S1 Table presents the online training schedule, while S1 Fig displays the online training and disease management modules.

The eIMCI allows CHWs to log in via WeChat using unique credentials to manage their patients. During visits, the platform guides CHWs to collect patient information on basic details, danger signs, common diseases, and nutrition. Once all required information is entered, the eIMCI platform automatically generates recommendations for disease classification, treatment, medical advice, and follow-up dates. Patient data is accessible only to the assigned CHW.

The control group received 4-day face-to-face training (S2 Table) and used paper-based IMCI.

### Outcomes

Primary outcome: accuracy of disease classification at patient level. Secondary outcomes: treatment accuracy, time for assessment/classification/treatment, and training test scores.

Each CHW assessed three sequentially allocated eligible patients, with classifications and treatments validated by a paediatric specialist. Field researchers ensured protocol adherence and blinded assessments. Accuracy was judged against the specialist’s diagnosis and treatment.

To mitigate potential bias arising from differential expectations among CHWs, all CHWs received standardized training and collected required data related to core IMCI principles under the supervision of the paediatric specialist and the field researchers for quality control. Notably, the only differences between the two groups were in the delivery format of training and the tools employed.

Training costs were compared based on direct expenses, including trainer labour, materials, venue, transportation, and meals. Costs for developing online modules and network access were excluded.

The IMCI training test was administered to all 103 trainees at the end of the course, using a standardized written exam scored out of 100.

The field researcher recorded the time required for classification and treatment, from initial communication to treatment completion.

### Sample size

A planned sample of 36 children per arm (treated by 12 CHWs, i.e., clusters, with 3 children per CHW) was designed to achieve 95% power (via Unpooled T-Test) to detect a 40% absolute increase in correct disease classification (from 20% in the control group—based on prior IMCI projects in similar Chinese settings—to 60% in the intervention group, a conservative estimate from pilot data). This calculation was performed for a cluster-randomized design (two independent proportions) using PASS 2021 software, assuming an intra-cluster correlation coefficient (ICC) of 0.05 for the primary outcome [29].

### Statistical analysis

Continuous variables were described using mean and SD, categorical variables as percentages. Relative risk (RR) for binary outcomes, mean differences (MD) for continuous outcomes, as well as their 95% confidence intervals (CIs), were estimated using Poisson and linear generalized linear mixed models (GLMM), respectively (i.e., Poisson GLMM for binary outcomes and linear GLMM for continuous outcomes), with a random intercept for CHW to account for clustering of children within CHWs. Model assumptions were rigorously evaluated prior to analysis: For Poisson GLMMs, overdispersion was assessed via the ratio of residual deviance to drees of freedom and a likelihood ratio test comparing the Poisson model with a negative binomial GLMM, confirming no significant overdispersion (p > 0.05). Unadjusted and adjusted results were reported separately based on whether the models were adjusted for covariates at both the CHW level (age, sex, education status) and the patient level (age, sex, number of diseases). The analysis followed the intention-to-treat (ITT) principle: all children were analysed according to their original CHW group assignment. Additionally, subgroup analyses were conducted for the primary outcome (correct disease classification) by incorporating interaction terms between the grouping variable and subgroup variables, including parent age (≥45, <45 years), parent gender, parent education level (Secondary school or lower, junior college or above), as well as child age (<2, 2–<4, 4–5 years), child gender, and number of diseases (1 disease, 2 and above). All statistical analyses were performed using STATA 16.0. A two-sided P value < 0.05 was considered statistically significant.

### Patient and public involvement

The eIMCI platform was designed to enhance the capabilities of CHWs, thereby directly benefiting the public through improved healthcare delivery. Local health bureau officers and CHWs were engaged early in the development of the eIMCI platform and assisted in implementing the training, intervention, and evaluation for both groups. CHWs and child caretakers participated through informed consent. The study focused on common childhood illnesses, reflecting the typical patient population in rural China.

### Ethical approval and consent to participate

The Biomedical Ethics Review Committee of Peking University approved the eIMCI study (approval number: IRB00001052-20072). All interviewees including CHWs and child patients were required to provide informed consent.

### Registration

This trial was retrospectively registered (ChiCTR2100042533) after participant recruitment, primarily due to COVID-19-related disruptions to fieldwork timelines. Amid the pandemic, we prioritized scientific rigor and ethical approval, which inadvertently delayed trial registration. The registered protocol matched the ethically approved version, with all primary and secondary outcomes prespecified and unchanged post-recruitment. Protocol adjustments (e.g., shifting evaluations to a local city hospital and abandoning real-world comparisons of service volumes and patient referrals) were driven by COVID-19-related constraints. Notably, these changes did not alter the prespecified outcomes or core study design, and the reported findings are not biased by the delayed registration.

## Results

### Participant recruitment and characteristics

**Fig 1:**
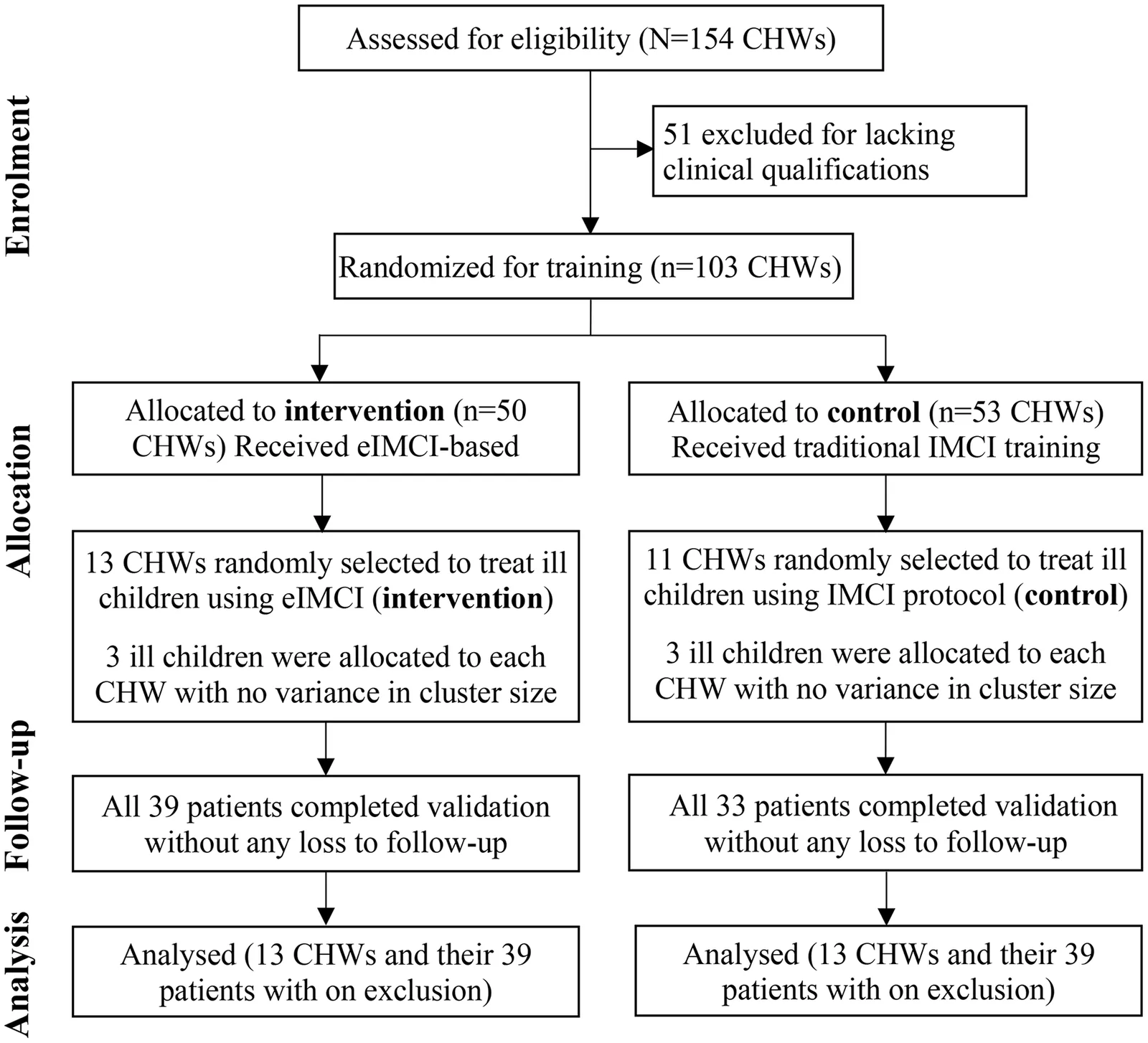
Participant flowchart.

A total of 103 CHWs were qualified and agreed to be randomly allocated to two training groups: traditional face-to-face IMCI training (53 CHWs) and eIMCI-based training (50 CHWs). The characteristics of all the 103 CHWs is shown in S3 Table. During the evaluation, one CHW in the control group failed to participate due to the need of COVID-19 control, while another CHW received the eIMCI training in the same township was sent to the city hospital instead. As a result, 39 eligible paediatric patients treated by 13 CHWs, and 33 patients treated by 11 CHWs were included in the intervention group and control group, respectively (This still has 95% power to test the expected difference. See Fig 1 for participant flowchart).

Among the 24 CHWs, 15 (62.5%) were male, and 13 (54.2%) were under 45 years old. For the participating children, 62.5% (45/72) were male, with an even distribution across age groups (<2 years, 2–4 years, and ≥4 years), and approximately half (52.8%, 38/72) had two or more diseases. No significant differences were observed in the baseline characteristics of participating CHWs or children between the intervention and control groups, except for a lower incidence of fever or otitis without infection in the intervention group (Table 1).

**Table 1:** Characteristics of assessed community health works and child patients.

Characteristics	Total		Control		Intervention	
	N	%	N	%	N	%
**Community health workers**	24	–	11	–	13	–
Age						
<45 years	13	54.2	5	45.5	8	61.5
≥45 years	11	45.8	6	54.6	5	38.5
Gender						
Female	9	37.5	3	27.3	6	46.2
Male	15	62.5	8	72.7	7	53.9
Education						
Secondary school or lower	19	79.2	8	72.7	11	84.6
Junior college or higher	5	20.8	3	27.3	2	15.4
**Paediatric patients**	72	–	33	–	39	–
Age						
2 months- <2 years	28	38.9	11	33.3	17	43.6
2- <4 years	20	27.8	7	21.2	13	33.3
≥4 years	24	33.3	15	45.5	9	23.1
Gender						
Female	27	37.5	11	33.3	16	41.0
Male	45	62.5	22	66.7	23	59.0
Number of diseases						
1	34	47.2	16	48.5	18	46.2
2 and above	38	52.8	17	51.5	21	53.9
Upper respiratory infections						
No	30	41.7	14	42.4	16	41.0
Yes	42	58.3	19	57.6	23	59.0
Fever						
No	31	43.1	10	30.3	21	53.9
Yes	41	56.9	23	69.7	18	46.2
Otitis without infection						
No	52	72.2	20	60.6	32	82.1
Yes	20	27.8	13	39.4	7	18.0
Diarrhoea						
No	58	80.6	26	78.8	32	82.1
Yes	14	19.4	7	21.2	7	18.0

Note: Community health workers are commonly called village doctor in rural China.

### Evaluation of disease management

CHWs using the eIMCI were more likely to correctly classify the children’s disease: 64.1% (25/39) in the intervention group, compared to 39.4% (13/33) in the control group (p = 0.056). After controlling for covariates, the results of the multivariable regression model showed that correct classification was twice as likely using eIMCI (RR = 2.1, 95%CI: 1.5–3.1, p < 0.001). (Table 2) Subgroup analyses of correct classification revealed no statistically significant heterogeneity across subgroups stratified by CHW-related or ill child-related variables (all interaction P-values > 0.05) (S4 Table). The ICC for accurate classification is < 0.001 at CHW level.

**Table 2:** Multivariable regression results of eIMCI intervention.

Outcome Variables	Intervention group	Control group	Unadjusted effect (95% CI)	Unadjusted p-value	Adjusted effect (95% CI) ¹	Adjusted p-value¹
**Binary Outcomes**						
**Correct disease classification**	25/39 (64.1%)	13/33 (39.4%)	RR = 1.6 (1.0, 2.7) ²	0.056	RR = 2.1 (1.5, 3.1)	<0.001
**Correct treatment**	15/39 (38.5%)	9/33 (27.3%)	RR = 1.4 (0.6, 3.5) ²	0.455	RR = 1.3 (0.6, 3.0)	0.526
**Continuous Outcomes**						
**Time for assessment, classification and treatment (min)**	11.3 ± 0.56 ³	13.8 ± 1.6 ³	MD = −2.5 (−5.7, 0.7) ⁴	0.064	MD = −3.4 (−6.8, 0.0)	0.053
**Training test scores**	60.9 ± 3.0 ³	62.7 ± 2.4 ³	MD = −1.9 (−10.1, 6.4) ⁴	0.320	MD = −2.9 (−11.6, 5. 8)	0.493

¹ Adjusted for CHW-level covariates (age, sex, education status) and patient-level covariates (age, sex, number of diseases), with clustering accounted for via random intercepts for CHWs.

² Relative risk (RR) for binary outcomes.

³ Mean ± standard error (SE) for normally distributed continuous outcomes.

⁴ Mean difference (MD) for normally distributed continuous outcomes.

No significant differences were observed between the intervention and control groups in terms of correct treatment. Specifically, the proportion of correct treatment was 38.5% (15/39) in the intervention group and 27.3% (9/33) in the control group (P = .455). Multivariable regression analysis also showed no significant difference between the groups (RR = 1.3, 95% CI: 0.6–3.0) (Table 2).

The mean time spent by CHWs on assessment, diagnosis, and treatment per child was 11.3 minutes (standard error [SE]: 0.56) in the intervention group and 13.8 minutes (SE: 1.6) in the control group (P = .064). In the adjusted multivariable regression model, the mean difference (MD) between groups was −3.4 minutes, approaching statistical significance (p = 0.053) (Table 2).

### Evaluation of training

The control group underwent a standard training program totalling 24 hours of onsite training (6 hours across 4 days), whereas the eIMCI online training averaged 5.2 hours (SE: 0.3) for all 50 trainees and 4.9 hours (SE: 0.7) for the 13 trial participants, yielding an 80% reduction in time. Despite this efficiency, post-training test scores for the 103 trainees were lower in the intervention group than in the control group, with average scores of 52.4 (SE: 2.2) versus 61.4 (SE: 1.3) (*P* < .001) (Table 3). The eIMCI training incurred a cost of CNY134 per CHW, which was substantially lower – 82.6% less – than the traditional offline training at CNY772 per CHW (Table 4).

**Table 3:** Training time and effect (test scores) among all 103 CHWs randomly allocated to the eIMCI (intervention) and IMCI (control) groups.

Indicators	Groups	N	Mean	SE	95% CI		P-value ^a^
Training time (hours/person)							
	eIMCI	50	5.2	0.7	4.7	5.8	<0.001
	IMCI	53	24	0	24	24	
Test score out of 100							
	eIMCI	50	52.4	2.2	48.0	56.8	<0.001
	IMCI	53	61.4	1.3	58.9	64.0	

Note: t-test was used to detect group differences.

**Table 4:** Comparison of direct costs (CNY) of offline and online IMCI training.

Items	Unit	Offline (53 CHWs)			Online (50 CHWs)		
		Unit cost	Unit number	Sub-total	Unit cost	Unit number	Sub-total
Trainer labour fee	Person day	1200	6	7200	800	3	2400
Costs for transportation and meals	Person day	100	212	21200	–	0	0
Training material cost	Set	70	53	3710	70	50	3500
Training venue cost	Day	2000	4	8000	–	0	0
Organization	Set	800	1	800	800	1	800
Total	–	–	–	40910	–	–	6700
Cost per capita	–	–	–	772	–	–	134

Note: The offline training lasted for 4 days, while the online training could be completed within one month; IMCI: Integrated Management of Childhood Illness; CHW: Community health worker (the trainees).

## Discussion

### Principal findings

This randomized controlled trial assesses the effectiveness and efficiency of eIMCI, an innovative approach that combines structured training with stepwise decision support for managing childhood illnesses. Our findings indicate that eIMCI significantly doubled the rate of correct disease classification compared to traditional IMCI, although improvements in disease treatment were not statistically significant. eIMCI’s online training cut training time and costs by approximately 80%, albeit with a slight reduction in post-training test scores, suggesting a minor compromise in training efficacy. Additionally, no significant difference was observed in the time spent on patient management between the groups.

### Comparison with prior work

Our study revealed that community health workers (CHWs) had lower correct classification and treatment rates for childhood illnesses compared to other studies. Despite standard IMCI training and monitoring by a paediatric expert, control group CHWs correctly classified and treated only 39.4% (13/33) and 27.3% (9/33) of patients, respectively. This contrasts with a systematic review reporting correct classification and treatment rates ranging from 33%−90% (mostly above 60%) and 11%−100% (mostly above 50%), respectively [7]. The lower rates in our study may stem from the children’s more complex health conditions and the CHWs’ insufficient training.

Two controlled trials assessed the impact of electronic IMCI on accurate disease classification and treatment without integrating training modules into their platforms, unlike our eIMCI [24,25]. Both trials affirmed eIMCI’s benefit in correct disease classification, yet the improvement in correct treatment was not significant. Specifically, a before-after cluster trial in Tanzania found electronic protocols more accurate in classification (90.9%) compared to paper-based IMCI (82.7%) (P < 0.001) [25], and a stepped-wedge cluster randomized trial in Burkina Faso noted a 79% versus 73% (*P* = 0.004) enhancement [24]. Despite their findings of eIMCI’s superiority, the improvements were less pronounced than in our study, possibly due to our lower baseline rates and unique aspects of our intervention. Moreover, a recent one-arm study in rural Bangladesh demonstrated that mHealth platforms might diminish CHWs’ training needs while preserving quality performance in assessing newborns’ danger signs [22].

Beyond improved disease management, eIMCI garnered positive feedback from users, particularly for reducing the cognitive load of memorizing complex algorithms. It also aids in creating structured electronic medical records and enables timely performance evaluations.

### Challenges in improving correct treatment

Improved diagnosis or disease classification underpins more accurate prescriptions/treatments but failed to drive significant improvement in correct treatment in this study, due to several factors. First, deep-rooted prescribing habits persist among HCWs, such as habitual antibiotic prescription, insufficient focus on newborn care, and inadequate guidance on infant feeding and nutrition. Second, the relatively low availability of essential medicines (e.g., amoxicillin and oral rehydration salts [ORS]) in routine practice further reinforces incorrect prescribing behaviours. Third, HCWs often show weak belief in guideline importance, overreliance on personal experience, lack of intrinsic motivation, and physical or cognitive overload—these factors align with those reported by Sarrassat S et al [24]. Additionally, three study-specific issues may contribute: eIMCI-based training is less effective than traditional face-to-face training (the latter offers more vivid teaching and hands-on practice to enhance prescribing experience and self-efficacy); baseline imbalances exist (e.g., more HCWs aged ≥45 years [54.6% vs. 38.5%], more male HCWs [72.7% vs. 53.9%], and more 4–5-year-old patients [45.5% vs. 23%] in the control group); and the small sample size limits statistical power to detect the improvement in treatment (RR = 1.3, 95% CI: 0.6–3.0), not a true absence of effect.

To address these challenges, targeted measures should focus on four key areas: First, tackle habitual behaviours and guideline adherence by integrating case-based discussions on irrational antibiotic use and nutrition guidance into regular HCW training, paired with periodic supervised feedback. Second, optimize essential medicine supply chains—collaborate with health systems to ensure consistent availability of key medicines at primary facilities, eliminating supply-related barriers to correct prescribing. Third, enhance eIMCI training efficacy by adding interactive modules (e.g., simulated consultations, video demonstrations) and post-training refreshers to match the effectiveness of face-to-face training. Fourth, improve future study design by expanding sample sizes to boost power for detecting treatment improvements and stratifying randomization by HCW age, gender, and patient age to avoid baseline imbalances.

### Training for childhood illnesses management

While eIMCI training reduces time and costs and offers flexible schedules, its lower post-training test scores (vs. in-person training) can be interpreted through digital learning theory. Per transactional distance theory [30], the lack of real-time instructor interaction in our static modules may have widened the “communication gap,” weakening knowledge absorption. Meanwhile, self-determination theory [31] suggests our program failed to support learner motivation—without instant feedback (for competence) or peer connection (for relatedness), engagement and test performance dropped.

These gaps may explain why the intervention group showed no significant improvement in treatment outcomes. To address this, we propose theory-aligned adjustments: (1) Add interactive elements (e.g., scenario simulations, gamified quizzes with instant feedback); (2) Include short synchronous virtual Q&As to reduce interaction gaps; (3) Supplement e-learning with hands-on practice to link theory and clinical use; (4) Offer annual refresher courses tailored to knowledge gaps. The web-based distance learning modules for IMCI recommended by the World Health Organization (WHO) should be incorporated [32].

### Future enhancement of eIMCI system

Based on our findings and other research, in addition to the above recommendations for improving correct treatment, eIMCI could be enhanced in several ways: (1) Develop algorithms that facilitate multi-user management, communication with households, performance evaluation, and integration with local systems and resources [8,33]. (2) Increase eIMCI’s adaptability to allow adept CHWs to bypass lengthy procedures without sacrificing care quality. (3) Update algorithms to improve the structured management of critically ill children and monitoring during pre-referral periods [34,35]. (4) Expand services and procedures for newborn illness management, early childhood development, and care for patients over 5 years old [36]. (5) Continue developing CHW capacity and updating training content based on the latest guidelines and resources. The relatively poorer online training performance observed in the eIMCI group may be partially attributed to community health workers’ (CHWs’) lower expectations or confidence regarding online training. Therefore, enhancing CHWs’ self-efficacy in online training should be considered as a strategy to further improve the quality of online training and optimize disease management practice.

Even with these improvements and free access to eIMCI, widespread adoption is not assured. It is crucial to consider CHWs’ working conditions, workflows, and competencies before implementation. Additionally, sustained health system support is vital for eIMCI’s successful rollout [37].

### Strengths and limitations

This study evaluated the accuracy and efficiency of an electronic version of IMCI which innovatively integrated structured training with stepwise decision support for managing childhood illnesses. Conducted in a low-income rural area of West China, the study’s setting and findings are relevant and potentially applicable to other low-income regions globally.

The limitations of this study are evident. First, the shift from community clinics to a hospital setting—necessitated by COVID-19 pandemic restrictions—undermines ecological validity and thus limits generalizability to real-world community practice. Even though we implemented targeted measures to mimic community conditions (e.g., equipping CHWs with examination tools standard in village clinics, such as thermometers and stethoscopes, while excluding hospital-specific equipment like blood gas analysers; instructing hospital staff to conceal diagnostic results, including lab reports and imaging findings, to ensure CHWs relied on their own assessments, consistent with their routine community workflows), two critical gaps remain that may prevent our findings from fully reflecting eIMCI’s performance in village clinics: children assessed in the hospital may have slightly more severe symptoms than those managed in community settings, and CHWs lacked access to community-specific resources—most notably the electronic doctor workstation systems routinely used in village clinics for real-time medical record-keeping and prescription.

Second, conducted in a rural area of western China with a small sample size (39 children in the intervention group, 33 in the control group), the study has limited generalizability beyond the study population—specifically to other regions or populations characterized by distinct healthcare challenges or cultural contexts. While the sample was statistically powered to detect differences in the primary outcome (correct disease classification), it was insufficient to: (1) Identify small-magnitude improvements in secondary outcomes (e.g., correct treatment)—improvements that could still be clinically meaningful in larger cohorts; (2) Conduct robust subgroup analyses (e.g., by CHW experience, child age, or disease severity) to pinpoint populations where eIMCI may deliver the greatest benefit; and (3) Account for unmeasured variability (e.g., regional differences in CHW training or clinic resource availability) that could impact eIMCI’s scalability.

Third, the COVID-19 pandemic precluded observation of the actual transfer/referral process for critically ill children and forced the abandonment of our original plan to compare service delivery volumes in a real-world community setting.

Finally, close monitoring by a paediatric specialist and researchers likely enhanced adherence to the IMCI protocol—but this effect may have been more pronouncedin the control group. Since eIMCI’s decision-support function is less susceptible to external influence, the heightened protocol adherence in the control group may have underestimated the true benefit of eIMCI.

## Conclusions

Our study demonstrated the potential of the eIMCI platform to assist primary healthcare workers in effectively managing common childhood illnesses. Additionally, the eIMCI platform offers advantages over traditional IMCI by reducing training duration and costs. However, further enhancements are needed to improve training quality assurance, integrate the platform with existing electronic medical records systems, and conduct real-world evaluations in primary care settings.

**Strengths and limitations of this study**

- The intervention (eIMCI) innovatively integrated structured training with stepwise decision support for managing childhood illnesses, offering a novel approach to healthcare delivery.
- The trial generally evaluated the accuracy of eIMCI in disease classification and treatment, as well as its efficiency in reducing training time and costs, and the time required for disease management during clinic visits.
- Conducted in a low-income rural area of West China, the intervention and findings provide insights for future studies in other low-income regions globally.
- The COVID-19 pandemic necessitated hospital-based evaluations of ill children, modelled after the practice in village clinics. This approach precluded the observation of actual transfers or referrals of critically ill children, warranting caution when extrapolating the results to real-world community practice.
- The sample size is small, which limits the comprehensive evaluation, especially for the secondary outcomes.

## Supporting information

S1 Protocol IMCI protocol v2.1 (CN). (PDF)

S2 Protocol IMCI protocol v2.1 (EN). (PDF)

S1 Checklist CONSORT 2010 checklist. (DOCX)

S1 Table Schedule for online eIMCI training. (DOCX)

S1 Fig Screenshots of training and disease management modules in the eIMCI system. (PDF)

S2 Table Schedule for onsite IMCI training. (DOCX)

S3 Table Basic characteristics of all 103 village doctors participating in the training. (DOCX)

S4 Table Subgroup analyses for the primary outcome. (DOCX)

## Abbreviations

CHW: Community health worker
eIMCI: Electronic version of IMCI
IMCI: Integrated Management of Childhood Illness.
