# Supplementary material for: Assessing the accuracy and efficiency of an electronic platform for managing childhood illnesses in rural China: A cluster randomized controlled trial
Source: PLoS One. 2026 Sep 15;21(9):e0352583. doi: 10.1371/journal.pone.0352583 (PMC13577394; doi:10.1371/journal.pone.0352583)
Supplement: Supplementary file 1 [file pone.0352583.s001.pdf]

电子化儿童疾病综合管理项目评价研究（eIMCI）

研究背景

救助儿童会（SCI）在中国境内的活动经历及历史沿革

该项目由 SCI 资助。SCI 在中国已有 25 年的历史了，并且仍然是在中国开展业务最广的国际非政府组织之一。SCI 与中国政府、民间社会组织、私营公司，多边机构和个人合作，致力于促进儿童的福利，并努力确保所有儿童，无论其背景或能力如何，都能获得优质的服务。

SCI 一直倡导儿童生存是健康计划的关键成果。自 2010 年以来，SCI 与当地卫生部门，一线卫生服务提供者和家庭密切合作，以改善卫生服务以及在中国偏远农村社区正确诊断和治疗儿童疾病的举措。目前，在新疆维吾尔自治区和田州的巴音郭勒蒙古族自治州和墨玉县实施了儿童疾病综合管理项目（IMCI），其目标是提高一线卫生工作者对儿童常见病的综合管理水平 （例如肺炎和腹泻）。

乔治全球健康研究院（TGI）在中国境内的活动经历及历史沿革

TGI 中国在将移动技术整合到疾病控制和社区医疗保健方面具有很强的能力，资源和多年的经验。作为全球领先的研究机构，TGI 中国于 2014 年成立了由高通公司（Qualcomm Wireless Reach）资助的中国移动医疗创新中心（CCmHI），旨在通过研究可负担的，可持续的移动医疗干预措施来改善初级医疗保健，这些干预措施针对该国的主要死亡与疾病原因。在五年的实践中，根据 CCmHI 开展了一系列 mHealth 研究计划，以促进在母婴健康（MCH），糖尿病管理和营养领域的医疗服务的可及性和可用性。

IMCI 项目背景

降低儿童死亡率和发病率是全球改善人口健康的关键。因此，降低新生儿死亡率一直是可持续发展目标（SDGs）的重要组成部分(1)。 在中国，近年由于社会经济的快速发展以及政府对提高医疗服务质量及覆盖面的投入，儿童和孕产妇的健康状况得到了显著改善。例如，2009 年至 2018 年，全国五岁以下儿童死亡率（U5MR）从 17‰降至 8.6‰(2)。然而，尽管取得了令人瞩目的进步，但在中国偏远农村地区仍有一些儿童死

于常见、可预防、可治疗的儿童疾病，例如肺炎和腹泻。2018 年，农村和城市的 5 岁以下儿童死亡率（U5MR）分别为 10.2‰和 4.4‰，东部、中部和西部地区的 5 岁以下儿童死亡率分别为 4.2‰、7.2‰和 12.7‰(3)。这些差异与多种社会经济和地理特征有关，表明中国不同地区在儿童医疗服务方面存在显著差距(4)。

农村地区儿童生存面临的主要障碍之一是医疗资源分配不平等，包括医疗工作者、医疗设施和优质服务的分配不均衡(5–7)。事实上，中国所面临的问题并非个例。儿童疾病综合管理（IMCI）是一种简化的儿童疾病管理常规指南，已被证明是降低 5 岁以下儿童死亡率、疾病和残疾，以及促进儿童生长发育最为常用且有效的策略(8,9)。IMCI 已被证实不仅能改善医疗服务的质量，还能提升医疗工作者的能力，尤其是在资源有限的环境中(10–12)。因此，IMCI 有潜力降低中国大陆农村和西部地区的儿童死亡率和发病率。

IMCI 项目的实施策略

IMCI 在全球范围内被用作降低死亡率、疾病和残疾并促进五岁以下儿童改善生长发育的一项重要战略。根据 WHO IMCI Handbook， IMCI 包含三个主要组成部分：（1）改善卫生工作者的病例管理技能，（2）改善整体卫生系统，以及（3）改善家庭和社区卫生习惯。在医疗机构中，IMCI 策略可促进对门诊环境中常见的儿童期疾病的准确识别、确保适当的治疗加强对看护人的咨询，并加快将重病儿童转诊至更高级别的医疗机构的速度。在家庭环境中，它促进寻求适当医疗护理的行为，改善营养和预防性护理以及正确使用处方的情况。

SCI 于 2014 年将 IMCI 引入新疆的墨玉县。从 2014 年到 2018 年，该项目的重点是培训一线卫生工作者，改善监管并开展基于社区的健康促进活动。截至 2018 年 9 月，我们的项目覆盖了 16 家乡镇卫生院和 124 家乡村诊所，并通过 149 名受过培训的一线卫生工作者惠及 15,000 多名儿童。该计划的最终目的是通过不断增强当地卫生系统的服务能力并改善项目现场社区成员的看护行为，通过 IMCI 在中国西部的工作模式，确保儿童免受可预防的疾病的伤害。为了实现这一目的，将收集和分析基层卫生设施，尤其是乡村卫生所电子化 IMCI 的证据和数据，并在项目合作方（包括地方政府卫生机构）之间进行讨论，以制定适合当地的 IMCI 实施方法，以解决西北地区常见儿童疾病问题。

近年来，移动通信和智能健康技术的快速发展深刻改变了临床服务和就医行为(13,14)。互联网和其他智能健康技术能够为医疗决策提供精准且高效的支持(15)。此外，自 2020 年初开始的新冠疫情在全球范围内广泛改变了人们的工作和学习模式，而在线学习在这种情况下越来越受到青睐(16)。因此，将常规的儿童疾病综合管理（IMCI）与互联网和智能健康技术相结合，对于更好地实现改善中国欠发达地区儿童健康和福祉的目标至关重要。

基于以上背景信息，本研究假设在传统 IMCI 上进行创新可能会减少卫生工作者的工作量和劳动力成本，并借助集成的数字化 IMCI 管理和在线学习系统来确保农村地区 IMCI 实施的质量提升和更快速扩展。为了验证这一假设，北京航空航天大学已经完成开发了 IMCI 电子化模块。该模块集成了数字 IMCI 案例管理、在线课程学习和监督功能，并已完成测试。为了确定该系统效果的影响因素，并通过协调员将自我学习与小组学习的效果进行比较，本研究项目团队将进行 IMCI 电子化模块的评价研究。为了确定电子化 IMCI 有效性和扩展使用的影响因素，本项目将在结项前在一份全面的正式报告中汇报相关结果。

研究目标

本研究将设置使用 IMCI 电子化模块的干预组及使用传统方式的对照组，以完成以下目标：

I.通过调查患病儿童医疗服务质量，以及与专业儿科医师复核医嘱（金标准）的匹配度，对比评估干预组与对照组 IMCI 的实施效果；

II.评价干预组与对照组在 IMCI 培训的实施及培训效果方面的差异；

III. 评价 IMCI 电子化模块的可接受度以及在新疆省内推广的可行性。

研究方法

该研究设计为整群随机对照试验（cRCT），并作为嵌入式培训、临床管理以及不同处理方法效果和效率评价的基础。

1. 培训效果评估

设计了一项随机对照研究，通过与传统培训方法进行比较，以评估基于 eIMCI（电子儿童疾病综合管理）的在线培训的有效性。

培训对象、样本量和随机化：研究对象为库尔勒市所有 150 名具备诊治儿童疾病资质的乡村医生。他们将被集中随机分为干预组和对照组。每个乡镇卫生院下属的村卫生室（通常每所卫生室仅有一名医生）将被随机平均分配到干预组或对照组。剩余的单个乡镇卫生院的医生或村卫生室将被编为一组，然后随机分配到干预组或对照组。

培训方法和评估：参与研究的对照组乡村医生将接受传统的线下培训（见表 1），而干预组的乡村医生将通过 eIMCI 平台接受在线培训（见表 2）。

表 1. 现场 IMCI 培训日程表（每天 6 小时）

时间		内容	材料
第 1 天	开幕式	日程安排、分组、目标与任务、政策、后勤及拍照	IMCI 手册
	上午	讲座：第 1 部分．引言	
		讲座：第 2 部分．2 个月至 5 岁儿童的评估与分类	
	下午	讲座：第 2 部分．2 个月至 5 岁儿童的评估与分类（续）	
第 2 天	上午	讲座：第 3 部分．治疗计划的制定<br>问答环节	
		讲座：第 4 部分．治疗方案的实施<br>问答环节	
	下午	讲座：第 5 部分．对家长的建议<br>问答环节	
第 3 天	上午	讲座：第 6 部分．1 天至 2 个月儿童的管理	
	下午	讲座：第 6 部分．1 天至 2 个月儿童的管理（续）<br>问答环节	
		讲座：第 7 部分．随访<br>问答环节	
第 4 天	上午	笔试	
	下午	问答环节，培训结束	

表 2. 在线 eIMCI 培训日程表

<table>
<tr><th>事件</th><th>工具</th><th>内容</th><th>时间</th><th>时长</th><th>材料</th></tr>
<tr><td rowspan="2">开幕式</td><td rowspan="2">钉钉 App</td><td>日程介绍</td><td rowspan="2">第 1 天</td><td rowspan="2">40 分钟</td><td>/</td></tr>
<tr><td>eIMCI 系统使用演示</td><td>在线 IMCI 手册</td></tr>
<tr><td rowspan="9">在线学习</td><td rowspan="10">微信 App</td><td>第 1 部分. 引言（第 1 章&第 2 章）</td><td rowspan="3">第 2 天</td><td>40 分钟</td><td rowspan="9">手册、图表和练习（在线</td></tr>
<tr><td>第 2 部分. 2 个月至 5 岁儿童的评估与分类（第 1 章-第 3 章）</td><td>36 分钟</td></tr>
<tr><td>第 2 部分. 2 个月至 5 岁儿童的评估与分类（第 4 章&第 5 章）</td><td>41 分钟</td></tr>
<tr><td>第 2 部分. 2 个月至 5 岁儿童的评估与分类（第 6 章-第 9 章）</td><td rowspan="3">第 3 天</td><td>31 分钟</td></tr>
<tr><td>第 3 部分. 治疗计划的制定（第 1 章-第 4 章）</td><td>45 分钟</td></tr>
<tr><td>第 4 部分. 治疗方案的实施（第 1 章-第 6 章）</td><td>60 分钟</td></tr>
<tr><td>第 5 部分. 对家长的建议（第 1 章-第 6 章）</td><td rowspan="4">第 4 天</td><td>45 分钟</td></tr>
<tr><td>第 6 部分. 1 天至 2 个月儿童的管理（第 1 章-第 3 章）</td><td>40 分钟</td></tr>
<tr><td>第 7 部分. 随访（第 10 章）</td><td>20 分钟</td></tr>
<tr><td>测试</td><td></td><td>60 分钟</td><td>/</td></tr>
</table>

注：培训项目由地方政府组织；培训师为县级医院的儿科医生。来自首都儿科研究所的国家级专业人员对县级培训师进行了培训（一级培训）。然后由县级培训师对乡村医生进行培训（二级培训）。在二级培训之前，要求县级培训师进行试点培训课程（质量控制）。IMCI 培训材料由首都儿科研究所根据世界卫生组织的标准套餐改编而成。

培训结束后，将向参与者发放统一的测试问卷以评估培训效果，并为每位受训者给出 0-100 分的测试成绩。

2. 临床管理效果评估

结局指标：主要结局指标是疾病分类的准确率（即症状和严重程度被正确分类的儿童所占比例）。次要结局指标是正确治疗率（即儿童被正确诊断和治疗的比例），以及指导用药的标准率（即向家长解释剂量和用药方法的比例）。疾病分类和治疗是否正确将由擅长儿科诊断和治疗技术的儿科专家通过现场检查和/或复测来判断。在医生的

日常诊疗过程中，熟悉 IMCI 技术的儿科专家将随机选择 3 名在选定的卫生院或诊所接受治疗的儿童，观察其诊疗过程和结果，并记录每名儿童疾病的诊断分类和治疗措施，评估其正确性。

研究对象：参加 IMCI 项目的乡镇卫生院和村卫生室的医生所诊治的 0 至 5 岁儿童患者。儿童纳入标准：（1）儿童患者年龄在 0 至 5 岁之间；（2）儿童的家长签署知情同意书；（3）儿童患有常见儿科疾病；以上三项必须同时满足才能纳入研究。排除标准：年龄超出 0 至 5 岁范围的儿童患者；儿童患者的家长未签署知情同意书；患有慢性、罕见儿科疾病、意外伤害等，其医生或家长认为 IMCI 诊疗可能对病情有害的儿童患者。

抽样和随机化：符合条件的患者将按顺序从在培训过程中被随机分为干预组和对照组的医生所治疗的患者中选出。在本研究中，一个“群组”是实施治疗（传统 IMCI 和 eIMCI）的最小单位——乡镇卫生院或村卫生室的一名医生。用于质量评估的研究医生将从受过培训的医生中集中随机选出。为了提高可比性并避免交叉污染，一个乡镇卫生院可以同时有干预组医生和对照组医生，但一个村卫生室即使有两名医生，也只属于一个组。在乡镇卫生院和村卫生室两个层面保持样本量一致的原则下，抽样时首先随机选出 6 个乡镇卫生院，每个乡镇卫生院各选出一名干预组医生和一名对照组医生；在所选乡镇卫生院下属的村卫生室中，再随机选出一个干预组村卫生室和一个对照组村卫生室。最终，共选出 24 名医生，包括 12 名乡镇医院医生（干预组和对照组各占一半）和 12 个村卫生室（干预组和对照组各占一半）。在每位医生/诊所的日常诊疗中，将按顺序随机选择 3 名儿童作为研究对象，并由儿科专家对乡镇和村卫生室医生的诊疗行为进行对比观察。

样本量和统计考虑：基于整群随机对照研究设计（村医为群，其诊疗的患儿为研究对象），1:1 随机分组，主要观察指标为儿童常见病的分类准确率。经过计算，可基于干预组 12 名医生（平均每名医生处理 3 例患儿）诊治的 36 例患儿，与对照组 12 名医生诊治的 36 例患儿，可有 95%的检验效能检测出疾病分类正确率 40%的绝对提升（对照组基线正确率基于中国相似场景下的既往 IMCI 项目数据设定为 20%，干预组目标正确率基于预试验数据保守估计为 60%）。此样本量估算基于整群随机设计两独立样本率比较，检验水准设为 5%（α=0.05），并假设每名医生分类准确率的内部一致性系数（ICC）为 0.05。两组间主要结局指标与次要结局指标的差异将采用广义线性混合模型（GLMM）进行比较，主模型将对聚类效应（以村医为聚类单位）及以下协变量进行校

正：医生层面变量（年龄、性别、教育程度）与患者层面变量（年龄、性别、患病种数）。

干预措施：对照组医生将使用传统的纸质 IMCI 流程完成儿童的诊断和治疗。干预组医生将在 eIMCI 决策支持平台的支持下完成儿童的诊断和治疗。

- 纸质 IMCI：标准化儿科适宜技术的诊断和治疗流程包括以下步骤：评估儿童、对疾病进行分类、治疗儿童、指导母亲如何继续治疗以及如何在家中自行提供适当的护理，以及管理随访(17)。这些步骤与目前用于治疗儿童的流程相似，但部分术语有所不同。评估是指询问病史和进行体格检查。分类是指确定儿童的症状和严重程度，并进行分类以提供治疗依据。治疗是指立即为儿童提供药物或其他治疗。指导母亲是指关于儿童喂养和后续随访的指导。IMCI 评估流程包括以下内容和步骤。首先，询问儿童就诊的原因并检查一般危险症状；询问四种主要症状：咳嗽或呼吸困难、腹泻、发热和耳部疾病，如果出现其中一种，则继续进行相关的下一步评估和分类。在这些步骤之后，医生应检查儿童是否患有营养不良，询问疫苗接种和维生素补充情况，并回答母亲的其他问题。
- eIMCI：eIMCI 由北京航空航天大学开发。模块集成了包括病例管理、在线课程学习和监督在内的多种功能，这些功能是基于常规 IMCI 的逻辑进行编制。

3. 工作效率评估

工作效率的评估是对接受两种培训和使用及部使用 eIMCI 的所有医生完成儿科诊断和治疗累计月门诊量的对比。这些数据可以通过当地卫生系统获得。

4. 可接受性评估

本研究部分将对当地卫生部门官员、乡镇卫生院领导和医生、乡村医生以及儿童家长进行定性访谈（根据具体情况选择面对面访谈或电话访谈），以分析在线培训对儿科诊断和治疗适宜技术的可接受性，以及使用小程序进行诊断和治疗的可接受性，以及在有需求的地区进一步推广的可行性。定性访谈部分将采用基于理论的方法，采用目的性抽样，使用基于行为理论框架起草的半结构化问卷进行数据收集和初步编码，并进行实时比较分析。访谈部分计划选择访谈对象，包括 12-20 名乡镇医生/乡村医生（干预组和对照组各占一半）、12-20 名儿童家长（干预组和对照组各占一半）、当地卫生部门工作人员和/或其他利益相关者约 6-10 人。在实际操作中，最终访谈人数将

根据信息饱和度确定。乡村医生和家长将从评估儿科诊断和治疗适宜技术有效性的调查对象中选择，不加区分。其他利益相关者，包括当地卫生系统工作人员，将从与乡村医生日常工作相关的人群中随机选择。

5. eIMCI 卫生经济学评估

将从卫生系统角度对成本以及每名儿童的成本进行全面分析。将总结、比较和评估干预组和对照组的成本构成、金额和项目实施效果。

通过成本效果分析和增量成本效益比方法进行卫生经济学评估，其中分子是干预组和对照组之间的费用差异，分母是两组所服务的患病儿童之间的差异。成本将基于项目培训师和材料的成本、参与培训的乡村卫生工作者产生的交通、住宿和误工费用以及软件维护和升级费用进行计算。效果将基于调查员收集的定量数据进行计算。增量效果是干预组和对照组正确疾病分类和治疗的儿童数量（比例）之间的差异。增量成本是干预组和对照组在儿童疾病综合管理培训（和/或平台开发和维护）相关成本之间的差异。

数据管理

来自乔治研究所的专业人员将建立数据库，监督数据收集，进行数据清理，并对项目的定量和定性数据进行分析。研究人员将遵循相关法律法规的标准程序输入和编码数据，以确保数据安全。研究人员将检查和清理定量数据，处理重复数据、空值、缺失数据等问题。定性数据将由合格的第三方供应商进行转录，并由经验丰富的研究人员进行编码，以尽量减少偏差和错误。

数据分析

定量数据分析将通过 SAS（企业版）进行。连续变量将用均值和标准差（正态分布）或百分位数（非正态分布）来描述，而分类变量则以百分比表示。连续变量的组间差异将通过 t 检验进行分析，分类变量将进行卡方检验。研究人员将使用多变量回归模型对 IMCI 项目实施效果的主要结果进行多变量分析，并根据个体和/或机构层面调整基线特征/因素。

研究人员将为一对一访谈和/或焦点小组访谈设计半结构化访谈指南。

伦理

项目团队将向北京大学医学院医学生物伦理委员会提交伦理批准申请，并确认只有在符合伦理委员会和相关法律的要求的情况下，与该项目相关的所有活动才能得到实

施。在获得伦理批准并由参与者签署同意书之前，不会开展任何项目活动。有关该项目的道德规范仅适用于项目协议中涉及的活动，该协议内容现已获得 TGI 中国和 SCI 的确认。

讨论

该项目将利用来自基层公共卫生服务机构的数据、来自家庭及社会环境的科学证据，并在项目利益相关者（包括当地儿童父母和政府卫生部门）之间进行讨论，以针对当地情况制定适当的高效的 IMCI 项目实施方式以及推广策略，以降低中国西北地区的常见儿童疾病发生，以及避免其导致的死亡。

资助

救助儿童会国际（英国）北京代表处

1. Liu L, Oza S, Hogan D, Chu Y, Perin J, Zhu J, et al. Global, regional, and national causes of under-5 mortality in 2000-15: an updated systematic analysis with implications for the Sustainable Development Goals. Lancet. 2016 Dec 17;388(10063):3027–35.

2. UNICEF. Trends in under-five mortality rate 2019 [Internet]. 2019. Available from: https://data.unicef.org/country/chn/

3. Liu Y, Kang L, He C, Miao L, Qiu X, Xia W, et al. Neonatal mortality and leading causes of deaths: a descriptive study in China, 2014-2018. BMJ Open. 2021 Feb 4;11(2):e042654.

4. Wang Y, Li X, Zhou M, Luo S, Liang J, Liddell CA, et al. Under-5 mortality in 2851 Chinese counties, 1996-2012: a subnational assessment of achieving MDG 4 goals in China. Lancet. 2016 Jan 16;387(10015):273–83.

5. Liang S, Macinko J, Yue D, Meng Q. The impact of the health care workforce on under-five mortality in rural China. Hum Resour Health. 2019 Mar 18;17(1):21.

6. Wang S, Ren Z. Spatial variations and macroeconomic determinants of life expectancy and mortality rate in China: a county-level study based on spatial analysis models. Int J Public Health. 2019 Jun;64(5):773–83.

7. Song S, Hou Z, Zhang L, Meng Q, Kawachi I. Can equitable distribution of health resources reduce under-five mortality rate? A cross-sectional study with multilevel analysis of rural counties in China. The Lancet. 2018 Oct 1;392:S58.

8. Gera T, Shah D, Garner P, Richardson M, Sachdev HS. Integrated management of childhood illness (IMCI) strategy for children under five. Cochrane Database Syst Rev. 2016 Jun 22;2016(6):CD010123.

9. Bryce J, el Arifeen S, Pariyo G, Lanata C, Gwatkin D, Habicht JP, et al. Reducing child mortality: can public health deliver? Lancet. 2003 Jul 12;362(9378):159–64.

10. Chopra M, Patel S, Cloete K, Sanders D, Peterson S. Effect of an IMCI intervention on quality of care across four districts in Cape Town, South Africa. Arch Dis Child. 2005 Apr;90(4):397–401.

11. Bishai D, Mirchandani G, Pariyo G, Burnham G, Black R. The cost of quality improvements due to integrated management of childhood illness (IMCI) in Uganda. Health Econ. 2008 Jan;17(1):5–19.

12. Nguyen DTK, Leung KK, McIntyre L, Ghali WA, Sauve R. Does integrated management of childhood illness (IMCI) training improve the skills of health workers? A systematic review and meta-analysis. PLoS One. 2013;8(6):e66030.

13. Baker SB, Xiang W, Atkinson IJIA. Internet of Things for Smart Healthcare: Technologies, Challenges, and Opportunities. IEEE Journals & Magazine | IEEE Xplore. 2017;5:26521–44.

14. Hilbert M, López P. The world’s technological capacity to store, communicate, and compute information. Science. 2011 Apr 1;332(6025):60–5.

15. Moreira MWL, Rodrigues JJPC, Korotaev V, Al-Muhtadi J, Kumar N. A Comprehensive Review on Smart Decision Support Systems for Health Care. IEEE Systems Journal. 2019;13(3):3536-45.

16. Adedoyin OB, Soykan E. Covid-19 pandemic and online learning: the challenges and opportunities. Interactive Learning Environments. 2020;31(2):1–13.

17. WHO, UNICEF. Handbook: IMCI integrated management of childhood illness [Internet]. [cited 2020 Apr 9]. Available from: https://iris.who.int/handle/10665/42939
