# Supplementary material for: Assessing the accuracy and efficiency of an electronic platform for managing childhood illnesses in rural China: A cluster randomized controlled trial
Source: PLoS One. 2026 Sep 15;21(9):e0352583. doi: 10.1371/journal.pone.0352583 (PMC13577394; doi:10.1371/journal.pone.0352583)
Supplement: Supplementary file 2 [file pone.0352583.s002.pdf]

Study on the evaluation of electronic Integrated Management of Childhood Illness (eIMCI)

Introduction

Decreasing child mortality and morbidity is key to improving population health globally. Therefore, reducing neonatal mortality has been an essential part of the Sustainable Development Goals (SDGs) (1). For China, the national under-five mortality rate (U5MR) has dropped from 17‰ to 8.6‰ between 2009 and 2018, showing that China has made much progress in improving children's health and wellbeing (2). However, the U5MR estimation in rural and urban area were 10.2‰ and 4.4‰, and that in the eastern, middle and western area were 4.2‰, 7.2‰ and 12.7‰ in 2018 (3). The variations were found associated with multiple socioeconomic and geographic characteristics, indicating a substantial gap in child healthcare services across different regions of China (4).

One of the major barriers to child survival in rural area is the inequality of healthcare resource distribution, including unbalanced allocation of healthcare workers, facilities, and quality services (5-7). Indeed, the difficulties China has encountered are not unique. The Integrated Management of Childhood illnesses (IMCI), a simplified routine guideline for child disease management, has been the most commonly used and effective strategy to reduce mortality, disease and disability, and to promote growth and development of children under five (8, 9). IMCI has been tested effective for improving not only the quality of healthcare service, but also the ability of health workers, especially in resource limited settings (10-12). Therefore, IMCI has the potential to decrease child mortality and morbidity in rural and western areas of mainland China.

In recent years, the rapid development of mobile communication and smart health technologies have profoundly changed clinical services and healthcare-seeking behaviors (13, 14). Internet and other smart health technologies can provide vast support to medical decision making with precision and efficiency (15). Moreover, the pandemic of COVID-19 from early 2020 has widely altered working and learning pattern of people globally, and online learning is now more and more preferred under the situation (16). Therefore, it is essential to combine the routine IMCI with internet and smart health technologies, to better achieve the goal of improving child health and wellbeing in under-developed regions of China.

Based on the above information, we hypothesize that an electronic IMCI (eIMCI) could improve the efficiency and quality of childhood disease diagnosis and management when compared to the standard paper-based IMCI. To test the hypothesis, this study aims to: (i) evaluate the outcome of clinical management (number and accuracy/correctness of childhood disease diagnosis and treatment) between the intervention group using an eIMCI and the control group using the paper-based IMCI, (ii) compare the outcome between an online training program delivered through eIMCI and the traditional training program, and (iii) evaluate acceptability of eIMCI by qualitative interview.

Methods

The study was designed as a cluster randomized controlled trial (cRCT), which was the basis for the evaluation of embedded training, the effectiveness in clinical management and the efficiency different treatments.

1. Evaluation of training

A randomized controlled study is designed to evaluate the effectiveness of the eIMCI-based online training by comparison with traditional training methods.

Trainees, sample size and randomization: The research objects are all 150 village doctors with qualifications for diagnosis and treatment of childhood diseases in Korla City. They will be centrally randomized into the intervention group and the control group, and the village clinics under each township health center (usually only one doctor) are evenly divided into the intervention group or the control group at random, and the left single township hospital doctors or village clinics are grouped together and then divided into the intervention group and the control group randomly.

Training methods and evaluation: Participating village doctors in the control group will receive the traditional offline training (Table 1) and those in the intervention group will receive an online training (Table 2) delivered through the eIMCI platform.

Table 1. Schedule for onsite IMCI training (6 hours per day)

**Time**		**Content**	**Material**
**Day 1**	Opening ceremony	Agenda, grouping, objectives and assignments, policies, logistics and photo shooting	IMCI handbook
	Morning session	**Lecture：** Section 1. Introduction	
		**Lecture：** Section 2. Evaluation and classification of children aged 2 months to 5 years	
	Afternoon session	**Lecture：** Section 2. Evaluation and classification of children aged 2 months to 5 years (continued)	
**Day 2**	Morning session	**Lecture：：** Section 3. Development of treatment plan<br>Q&A	
		**Lecture：** Section 4. Treatment delivery<br>Q&A	
	Afternoon session	**Lecture：** Section 5. Advices to the parents<br>Q&A	
**Day 3**	Morning session	**Lecture：** Section 6. Management of children aged 1 day to 2 months	
	Afternoon session	**Lecture:** Section 6. Management of children aged 1 day to 2 months (continued)<br>Q&A	
		**Lecture：** Section 7. Follow-up visit<br>Q&A	
**Day 4**	Morning session	Written exam	
	Afternoon session	Q&A, end of training	

Table 2. Schedule for online eIMCI training

Event	Tool	Content	Time	Duration	Material
Opening ceremony	Dingding (app)	Introduction to the agenda	Day 1	40 min	/
		Demonstration of how to use eIMCI system			IMCI handbook (online)
Online study	WeChat app	Section 1. Introduction (Chapter 1&2)	Day 2	40 min	Handbook, charts, and exercise (online)
		Section 2. Evaluation and classification of children aged 2 months to 5 years (Chapter 1-3)		36 min	
		Section 2. Evaluation and classification of children aged 2 months to 5 years (Chapter 4&5)		41 min	
		Section 2. Evaluation and classification of children aged 2 months to 5 years (Chapter 6-9)	Day 3	31 min	
		Section 3. Development of treatment plan (Chapter 1-4)		45 min	
		Section 4. Treatment delivery (Chapter 1-6)		60 min	
		Section 5. Advice to the parents (Chapter 1-6)	Day 4	45 min	
		Section 6. Management of children aged 1 day to 2 months (Chapter 1-3)		40 min	
		Section 7. Follow-up visit (Chapter 10)		20 min	
Test		Written exam		60 min	/

Note: The training program was organized by local governments; the trainers were doctors of pediatrics at county-level hospitals. National level professionals from Capital Institute of Pediatrics trained the county-level trainers (Primary level). Then the county-level trainers trained the village doctors (Secondary level). Before second-level training, the county-level trainers were asked to perform pilot training course (Quality control). IMCI training materials were generated by Capital Institute of Pediatrics adapted from WHO standard package.

A uniformed test questionnaire will be given to test the training effect after training, and a test score of 0-100 will be given for each trainee.

2. Clinical management evaluation

Outcomes: The primary outcome is the rate of correct disease classification (that is, the proportion of children with symptoms and severity to be correctly classified). Secondary outcome is the correct treatment rate (that is, the proportion of correct diagnosis and treatment for children), and the standard rate of guiding medication (the proportion of explaining the dosage and medication method to parents). Whether the disease classification and treatment

are correct will be judged by paediatricians who are expert at paediatric diagnosis and treatment techniques through on-site inspection and/or retest. During doctors' daily diagnosis and treatment, the paediatric experts who are also familiar with IMCI technique will review the process and results of 3 children in selected health centers or clinics without selection, and will record the diagnosis classification and treatment measures of each child's disease, and evaluate the correctness.

Research subjects: children patient aged 0 to 5 years who are treated by doctors in all township hospitals and village clinics participating in the IMCI project. Inclusion criteria for children: (1) The children patients are aged within the range of 0 to 5 years; (2) The parent of the child signs an informed consent form; (3) The child has usual pediatric diseases; the above three items must be met at the same time to be included in the study. Exclusion criteria: children patient aged beyond period of 0 to 5; Parents of the child patient do not sign the informed consent form; Children patient has chronic, unusual pediatric diseases, accidental injury and so on that their doctors or parents think the IMCI visit might do harm to the conditions.

Sampling and randomization: Eligible patients will be sequentially selected from the patients treated by doctors who have been randomly separated into the intervention and control group during the training process. In this study, a cluster is the smallest unit for the implementation of treatments (traditional IMCI and eIMCI) – a doctor in township hospital or village clinic. Study doctors for quality assessment will be centrally randomly selected from the trained doctors. To improve comparability and avoid contamination, a township health center can have both intervention group doctor and control group doctor, but a village clinic, even if there are two doctors, belong to one group. In the principle of consistency in sample size at the two levels of township health centers and village clinics, when sampling, 6 township health centers shall be first randomly selected, with one intervention group doctor and control group doctor from each township health center; among the selected village clinics under the township health centers, one intervening village clinic and one control village clinic are then randomly selected. In the end, a total of 24 doctors are selected, including 12 township hospital doctors (fifty-fifty for intervention and control) and 12 village clinics (fifty-fifty for intervention and control). During the daily diagnosis and treatment of each doctor/ clinic, three children shall be selected in order without selection as the research objects, and reviewed by pediatric experts with the diagnosis and treatment behaviors of the doctors in the township and village clinics.

Sample size and statistical considerations: The disease classification accuracy rate between 36 child patients treated by 12 doctors in the intervention group (on average 3 children per doctor, best for 2-4) and 36 patients treated by 12 doctors in the control group will be compared, and there should be 95% power (via Unpooled T-Test) to detect a 40% absolute increase in correct disease classification (from 20% in the control group—based on prior IMCI projects in similar Chinese settings—to 60% in the intervention group, a conservative estimate from pilot data). This estimation was performed for a cluster-randomized design (two independent proportions) at 5% significant level and based on 0.05 internal consistency (ICC) of each doctor's classification accuracy. The difference in the primary and secondary indicators between the two groups will be compared using the using generalized linear mixed models (GLMM) model, and the primary model will be corrected for the cluster (village doctors) and variables at the doctor level (age, gender, education status) and at the patient level (age, gender, number of diseases).

Treatments: Doctors in the control group will use the traditional paper-based IMCI route to complete the diagnosis and treatment of children. Doctors in the intervention group will complete the diagnosis and treatment of children with the support of the eIMCI decision support platform.

- **The paper-based IMCI*:*** The diagnostic and treatment process of standardized pediatric appropriate technology includes the following steps: evaluating the child, classifying the disease, treating the child, counseling the mother how to continue the treatment and provide appropriate care by herself at home, and managing the follow-up visit (17). These steps are similar to those currently used to treat children, but some terminology is different. Evaluating means asking for illness history and body check. Classifying refers to the determination of a child's symptoms and severity, and classification to provide evidence for treatment. Treating means to give medicine or other treatment to a child immediately and. Counseling the mother refers to guidance of child's feedings and subsequent visit. IMCI evaluating process includes following content and steps. Firstly, ask the reason why the child visit and check the general risk symptoms; and inquiry about four main symptoms: cough or difficulty breathing, diarrhea, fever and ear disease, and if one of them appears, continue the next relevant evaluation and classification. After these steps, doctors should check the child has malnutrition or not, inquiry vaccinations and vitamin supplements, and evaluate the mother's other questions.

- **The eIMCI:** The eIMCI was developed by Beihang University. The modules have integrated multiple functions including case management, online course learning, and supervision, which are adapted from routine IMCI protocol.

3. The efficiency evaluation

The evaluation index of the efficiency was the cumulative monthly outpatient visits of all rural doctors (about 150) in the intervention group and the control group who have completed training in appropriate techniques for paediatric diagnosis and treatment. The data could be obtained through the local health system.

4.Acceptability assessment

This part of the research will conduct qualitative interviews (face-to-face interviews or telephone interviews, depending on the specific situation) with local health department officials, township health center leaders and doctors, village doctors, and parents of children to analyze online training on appropriate techniques for pediatric diagnosis and treatment And the acceptability of using mini-programs for diagnosis and treatment, and the feasibility of further promotion in areas where there is demand. The qualitative interview part will adopt grounded theoretical methods, adopt intentional sampling, use semi-structured questionnaires drafted from the framework of behavioral theory for data collection and preliminary coding, and conduct real-time comparative analysis. The interview part plans to select interview subjects including 12-20 township doctors/village doctors (half the intervention group and the control group), 12-20 parents of the children (half the intervention group and the control group), local health department staff and/ Or about 6-10 other stakeholders. In actual operation, the final number of interviewees will be determined based on information

saturation. Rural doctors and parents will choose from the survey subjects for evaluating the effectiveness of appropriate techniques for pediatric diagnosis and treatment without distinction. Other stakeholders, including staff in the local health system, will be selected randomly from those connected with the daily work of village doctors.

5.eIMCI health economic evaluation

A comprehensive analysis of cost, and cost per child from the perspective of the health system will be conducted. The cost composition, amount, and project implementation effects of the intervention group and the control group will be summarized, compared, and evaluated. Health economics evaluation will be conducted through cost-effectiveness analysis and incremental cost-benefit ratio methods, where the numerator will be the difference in expenses between the intervention group and the control group, and the denominator will be the difference between the two groups of sick children served. The cost will be calculated based on the cost of project trainers and materials, the transportation, accommodation, and missed work expenses incurred by village health workers participating in the training, as well as the software maintenance and upgrade expenses. The effect will be calculated based on the quantitative data collected by the investigator. The incremental effect is the difference between the number (proportion) of children with correct disease classification and treatment in the intervention group and the control group. Incremental cost is the difference between the intervention group and the control group's child disease comprehensive management training (and/or platform development and maintenance) related costs.

Data management

Professionals from George Institute will establish database, supervise data collection, perform data cleaning and analyze the quantitative and qualitative data of the project. Researchers shall follow the standardized procedures of relevant laws and regulations to enter and encode data to keep data safe. Researchers will check and clean up quantitative data to deal with duplicate data, null values, missing data, etc. The qualitative data will be transcribed by qualified third-party suppliers and encoded by experienced researchers to minimize deviations and errors.

Data analyses

Quantitative data analysis will be done via SAS (Enterprise Edition). Continuous variables will be described in terms of mean and standard deviation (normal distribution) or percentiles (non-normal distribution), while categorical variables are expressed as percentages. The between-group difference of continuous variables will pass the t test, and the categorical variables will undergo the chi-square test. Researchers will use a multivariate regression model to conduct a multivariate analysis of the main results of the IMCI project implementation effect, and adjust the baseline characteristics/factors based on the individual and/or institutional level. Researchers will design semi-structured interview guidelines for one-on-one and/or focus group interviews.

Funding

Save the Children International (UK) Beijing Representative Office

References:

1. Liu L, Oza S, Hogan D, Chu Y, Perin J, Zhu J, et al. Global, regional, and national causes of under-5 mortality in 2000–15: an updated systematic analysis with implications for the Sustainable Development Goals. 2016;388(10063):3027-35.
2. UNICEF. Mortality rate, under-5 (per 1,000 live births) - China [Available from: https://data.worldbank.org/indicator/SH.DYN.MORT?locations=CN.
3. Liu Y, Kang L, He C, Miao L, Qiu X, Xia W, et al. Neonatal mortality and leading causes of deaths: a descriptive study in China, 2014–2018. 2021;11(2):e042654.
4. Wang Y, Li X, Zhou M, Luo S, Liang J, Liddell CA, et al. Under-5 mortality in 2851 Chinese counties, 1996–2012: a subnational assessment of achieving MDG 4 goals in China. 2016;387(10015):273-83.
5. Liang S, Macinko J, Yue D, Meng QJHrfh. The impact of the health care workforce on under-five mortality in rural China. 2019;17(1):1-13.
6. Wang S, Ren ZJIjoph. Spatial variations and macroeconomic determinants of life expectancy and mortality rate in China: a county-level study based on spatial analysis models. 2019;64(5):773-83.
7. Song S, Hou Z, Zhang L, Meng Q, Kawachi IJTL. Can equitable distribution of health resources reduce under-five mortality rate? A cross-sectional study with multilevel analysis of rural counties in China. 2018;392:S58.
8. Gera T, Shah D, Garner P, Richardson M, Sachdev HSJCDoSR. Integrated management of childhood illness (IMCI) strategy for children under five. 2016(6).
9. Bryce J, El Arifeen S, Pariyo G, Lanata CF, Gwatkin D, Habicht J-P, et al. Reducing child mortality: can public health deliver? 2003;362(9378):159-64.
10. Amaral JJFd, Victora CGJRBdSMI. The effect of training in Integrated Management of Childhood Illness (IMCI) on the performance and healthcare quality of pediatric healthcare workers: a systematic review. 2008;8(2):151-62.
11. Chopra M, Patel S, Cloete K, Sanders D, Peterson SJAodic. Effect of an IMCI intervention on quality of care across four districts in Cape Town, South Africa. 2005;90(4):397-401.
12. Bishai D, Mirchandani G, Pariyo G, Burnham G, Black RJHe. The cost of quality improvements due to integrated management of childhood illness (IMCI) in Uganda. 2008;17(1):5-19.
13. Baker SB, Xiang W, Atkinson IJIA. Internet of things for smart healthcare: Technologies, challenges, and opportunities. 2017;5:26521-44.
14. Hilbert M, López P. The World's Technological Capacity to Store, Communicate, and Compute Information. 2011;332(6025):60-5.
15. Moreira MWL, Rodrigues JJPC, Korotaev V, Al-Muhtadi J, Kumar N. A Comprehensive Review on Smart Decision Support Systems for Health Care. IEEE Systems Journal. 2019;13(3):3536-45.
16. Adedoyin OB, Soykan E. Covid-19 pandemic and online learning: the challenges and opportunities. Interactive Learning Environments. 2020:1-13.
17. WHO, UNICEF. Handbook IMCI: integrated management of childhood illness: World Health Organization; 2005.
