# Supplementary material for: Assessing the accuracy and efficiency of an electronic platform for managing childhood illnesses in rural China: A cluster randomized controlled trial
Source: PLoS One. 2026 Sep 15;21(9):e0352583. doi: 10.1371/journal.pone.0352583 (PMC13577394; doi:10.1371/journal.pone.0352583)
Supplement: Supplementary file 3 [file pone.0352583.s005.pdf]

S1 Fig. Screenshots of training and disease management modules in the eIMCI system

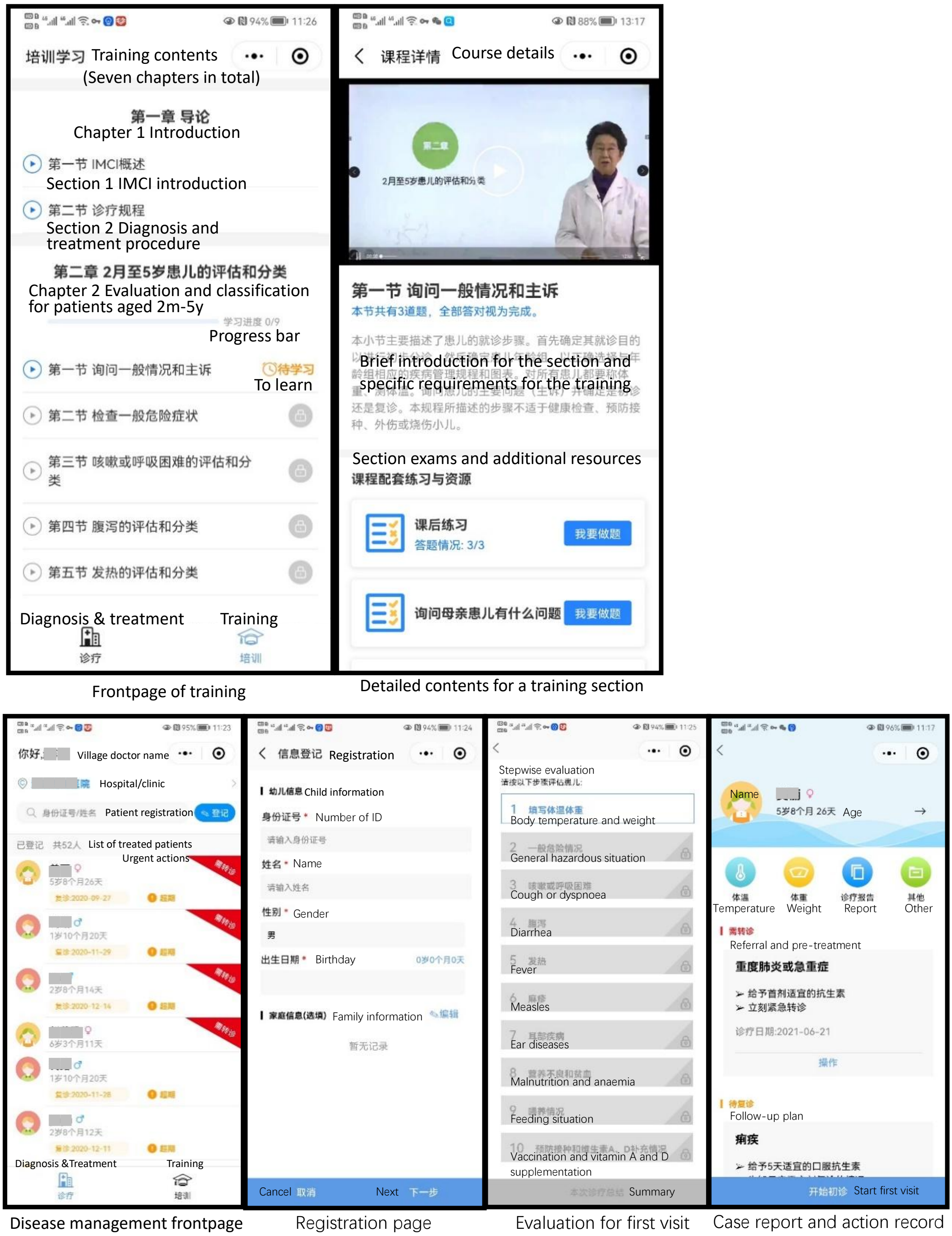

Note: The eIMCI system can provide structured online training and decision support for disease management including data entry, disease classification and treatment. When all classification and evaluation modules are completed, the eIMCI system will automatically produce diagnosis and treatment as well as medical advice and follow-up visit recommendations.
