# Supplementary material for: Assessing the accuracy and efficiency of an electronic platform for managing childhood illnesses in rural China: A cluster randomized controlled trial
Source: PLoS One. 2026 Sep 15;21(9):e0352583. doi: 10.1371/journal.pone.0352583 (PMC13577394; doi:10.1371/journal.pone.0352583)
Supplement: Supplementary file 5 [file pone.0352583.s004.docx]

# **S1 Table. Schedule for online eIMCI training**

| **Event** | **Tool** | **Content** | **Time** | **Duration** | **Material** |
| --- | --- | --- | --- | --- | --- |
| Opening ceremony | Dingding (app) | Introduction to the agenda | Day 1 | 40 min | / |
|  |  | Demonstration of how to use eIMCI system |  |  | IMCI handbook (online) |
| Online study | WeChat app | Section 1. Introduction (Chapter 1&2) | Day 2 | 40 min | Handbook, charts, and exercise (online) |
|  |  | Section 2. Evaluation and classification of children aged 2 months to 5 years (Chapter 1-3) |  | 36 min |  |
|  |  | Section 2. Evaluation and classification of children aged 2 months to 5 years (Chapter 4&5) |  | 41 min |  |
|  |  | Section 2. Evaluation and classification of children aged 2 months to 5 years (Chapter 6-9) | Day 3 | 31 min |  |
|  |  | Section 3. Development of treatment plan (Chapter 1-4) |  | 45 min |  |
|  |  | Section 4. Treatment delivery (Chapter 1-6) |  | 60 min |  |
|  |  | Section 5. Advice to the caretakers (Chapter 1-6) | Day 4 | 45 min |  |
|  |  | Section 6. Management of children aged 1 day to 2 months (Chapter 1-3) |  | 40 min |  |
|  |  | Section 7. Follow-up visit (Chapter 10) |  | 20 min |  |
| Test |  | Written exam |  | 60 min | / |

Note: The training program was organized by local governments; the trainers were doctors of pediatrics at county-level hospitals. National level professionals from Capital Institute of Pediatrics trained the county-level trainers (Primary level). Then the county-level trainers trained the community health workers (Secondary level). Before second-level training, the county-level trainers were asked to perform pilot training course (Quality control). IMCI training materials were generated by Capital Institute of Pediatrics adapted from WHO standard package.
