# Supplementary material for: Assessing the accuracy and efficiency of an electronic platform for managing childhood illnesses in rural China: A cluster randomized controlled trial
Source: PLoS One. 2026 Sep 15;21(9):e0352583. doi: 10.1371/journal.pone.0352583 (PMC13577394; doi:10.1371/journal.pone.0352583)
Supplement: Supplementary file 6 [file pone.0352583.s006.docx]

# **S2 Table. Schedule for onsite IMCI training**

| **Time** | | **Content** | **Material** |
| --- | --- | --- | --- |
| **Day 1** | Opening ceremony | Agenda, grouping, objectives and assignments, policies, logistics and photo shooting | IMCI handbook |
|  | Morning session | **Lecture：**Section 1. Introduction |  |
|  |  | **Lecture：**Section 2. Evaluation and classification of children aged 2 months to 5 years |  |
|  | Afternoon  session | **Lecture：**Section 2. Evaluation and classification of children aged 2 months to 5 years (continued) |  |
| **Day 2** | Morning session | **Lecture:：**Section 3. Development of treatment plan  Q&A |  |
|  |  | **Lecture：** Section 4. Treatment delivery  Q&A |  |
|  | Afternoon session | **Lecture：**Section 5. Advice to the caretakers  Q&A |  |
| **Day 3** | Morning session | **Lecture：**Section 6. Management of children aged 1 day to 2 months |  |
|  | Afternoon session | **Lecture:** Section 6. Management of children aged 1 day to 2 months (continued)  Q&A |  |
|  |  | **Lecture：**Section 7. Follow-up visit  Q&A |  |
| **Day 4** | Morning session | Written exam |  |
|  | Afternoon session | Q&A, end of training |  |
