# Supplementary material for: Assessing the accuracy and efficiency of an electronic platform for managing childhood illnesses in rural China: A cluster randomized controlled trial
Source: PLoS One. 2026 Sep 15;21(9):e0352583. doi: 10.1371/journal.pone.0352583 (PMC13577394; doi:10.1371/journal.pone.0352583)
Supplement: Supplementary file 7 [file pone.0352583.s007.docx]

# **S3 Table. Basic characteristics of all 103 village doctors participating in the training**

| **Variables** | **Total** | | **Traditional training** | | **eIMCI-based training** | | **P-value** |
| --- | --- | --- | --- | --- | --- | --- | --- |
|  | N | % | N | % | N | % |  |
| **Age (year)** |  |  |  |  |  |  |  |
| <45 | 69 | 66.99 | 36 | 67.92 | 33 | 66.00 | 0.836 |
| ≥55 | 34 | 33.01 | 17 | 32.08 | 17 | 34.00 |  |
| **Sex** |  |  |  |  |  |  |  |
| Female | 60 | 58.25 | 34 | 64.15 | 26 | 52.00 | 0.211 |
| Male | 43 | 41.75 | 19 | 35.85 | 24 | 48.00 |  |
| **Education** |  |  |  |  |  |  |  |
| Technical Secondary school | 69 | 66.99 | 36 | 67.92 | 33 | 66.00 | 0.836 |
| Junior college or above | 34 | 33.01 | 17 | 32.08 | 17 | 34.00 |  |
| **Distance from city** |  |  |  |  |  |  |  |
| <5km | 11 | 10.68 | 5 | 9.43 | 6 | 12.00 | 0.563 |
| 5-10 km | 15 | 14.56 | 10 | 18.87 | 5 | 10.00 |  |
| 10-30km | 59 | 57.28 | 28 | 52.83 | 31 | 62.00 |  |
| ≥50km | 18 | 17.48 | 10 | 18.87 | 8 | 16.00 |  |
| **Total** | 103 | 100.00 | 53 | 100.00 | 50 | 100.00 |  |

IMCI: Integrated Management of Childhood Illness
