# Supplementary material for: Assessing the accuracy and efficiency of an electronic platform for managing childhood illnesses in rural China: A cluster randomized controlled trial
Source: PLoS One. 2026 Sep 15;21(9):e0352583. doi: 10.1371/journal.pone.0352583 (PMC13577394; doi:10.1371/journal.pone.0352583)
Supplement: Supplementary file 8 [file pone.0352583.s008.docx]

**S4 Table.** Subgroup analyses for primary outcome (correct disease classification)

| **Subgroups** | **N** | **n** | **RR (95% CI)** | **P for interaction** |
| --- | --- | --- | --- | --- |
| **Village doctor level** |  |  |  |  |
| **Age** |  |  |  |  |
| **≥45 versus** | 39 | 20 | 2.53 (1.47, 4.35) | 0.640 |
| **<45 years** | 33 | 18 | 2.44 (1.29, 4.59) |  |
| **Gender** |  |  |  |  |
| Female | 27 | 13 | 2.41 (1.08, 5.37) | 0.547 |
| Male | 45 | 25 | 1.97 (1.16, 3.37) |  |
| **Education** |  |  |  |  |
| Junior college or above | 15 | 9 | 2.00 (2.00, 2.00) | 0.151 |
| Technical secondary school | 57 | 29 | 2.24 (1.43, 3.53) |  |
| **Child patient level** |  |  |  |  |
| **Age** |  |  |  |  |
| 2 months-<2 years | 28 | 14 | 2.58 (1.38, 4.83) | 0.576 |
| 2-<4 years | 20 | 10 | 1.72 (0.61, 4.86) |  |
| 4-5 years | 24 | 14 | 2.58 (1.14, 5.83) |  |
| **Gender** |  |  |  |  |
| Female | 27 | 13 | 1.28 (0.66, 2.48) | 0.081 |
| Male | 45 | 25 | 3.01 (1.58, 5.75) |  |
| **Number of diseases** |  |  |  |  |
| I disease | 34 | 27 | 1.64 (1.29, 2.08) | 0.159 |
| 2 and above | 38 | 11 | 3.84 (1.07, 13.86) |  |
